# A retrospective analysis of oral cholera vaccine use, disease severity and deaths during an outbreak in South Sudan

**DOI:** 10.2471/BLT.15.166892

**Published:** 2016-06-14

**Authors:** Cavin Epie Bekolo, Joris Adriaan Frank van Loenhout, Jose Manuel Rodriguez-Llanes, John Rumunu, Otim Patrick Ramadan, Debarati Guha-Sapir

**Affiliations:** a Ministry of Public Health, Centre Médical d’Arrondissement de Baré, Nkongsamba, Cameroon.; b Centre for Research on the Epidemiology of Disasters, Institute of Health and Society, Université Catholique de Louvain, 30 Clos Chapelle-aux-Champs, Brussels, 1200, Belgium.; c Ministry of Health, Juba, South Sudan.

## Abstract

**Objective:**

To determine whether pre-emptive oral cholera vaccination reduces disease severity and mortality in people who develop cholera disease during an outbreak.

**Methods:**

The study involved a retrospective analysis of demographic and clinical data from 41 cholera treatment facilities in South Sudan on patients who developed cholera disease between 23 April and 20 July 2014 during a large outbreak, a few months after a pre-emptive oral vaccination campaign. Patients who developed severe dehydration were regarded as having a severe cholera infection. Vaccinated and unvaccinated patients were compared and multivariate logistic regression analysis was used to identify factors associated with developing severe disease or death.

**Findings:**

In total, 4115 cholera patients were treated at the 41 facilities: 1946 (47.3%) had severe disease and 62 (1.5%) deaths occurred. Multivariate analysis showed that patients who received two doses of oral cholera vaccine were 4.5-fold less likely to develop severe disease than unvaccinated patients (adjusted odds ratio, aOR: 0.22; 95% confidence interval, CI: 0.11–0.44). Moreover, those with severe cholera were significantly more likely to die than those without (aOR: 4.76; 95% CI: 2.33–9.77).

**Conclusion:**

Pre-emptive vaccination with two doses of oral cholera vaccine was associated with a significant reduction in the likelihood of developing severe cholera disease during an outbreak in South Sudan. Moreover, severe disease was the strongest predictor of death. Two doses of oral cholera vaccine should be used in emergencies to reduce the disease burden.

## Résumé

**Objectif:**

Déterminer si la vaccination orale préventive contre le choléra diminue la gravité de cette maladie et la mortalité qui lui est associée chez les individus qui attrapent le choléra lors d'une flambée.

**Méthodes:**

L'étude s'est basée sur une analyse rétrospective de données démographiques et cliniques provenant de 41 centres de traitement du choléra au Soudan du Sud et relatives à des patients ayant contracté le choléra entre le 23 avril et le 20 juillet 2014 lors d'une épidémie de grande ampleur, quelques mois après une campagne de vaccination orale préventive. Les patients qui ont souffert de déshydratation sévère ont été considérés comme atteints d'une infection cholérique grave. Une comparaison a été établie entre les patients vaccinés et ceux qui ne l'ont pas été et une analyse de régression logistique multivariée a été utilisée pour identifier les facteurs associés au développement d'une forme sévère de la maladie ou au décès.

**Résultats:**

Au total, 4115 patients atteints de choléra ont été traités dans les 41 centres: 1946 (47,3%) ont développé une forme sévère de la maladie et 62 (1,5%) sont décédés. L'analyse multivariée a montré que le risque de développer une forme sévère de la maladie était 4,5 fois moindre pour les patients ayant reçu deux doses de vaccins oraux contre le choléra que pour les patients non vaccinés (rapport des cotes ajusté, RCa: 0,22; intervalle de confiance, IC, à 95%: 0,11–0,44). En outre, le risque de décès était considérablement plus élevé chez les patients ayant développé une forme sévère de la maladie que chez les autres (RCa: 4,76; IC à 95%: 2,33–9,77).

**Conclusion:**

La vaccination préventive avec deux doses de vaccins oraux contre le choléra a été associée à une réduction significative du risque de développer une forme sévère du choléra lors d'une flambée au Soudan du Sud. Par ailleurs, le développement d'une forme sévère de la maladie est le plus important facteur prédictif de décès. Deux doses de vaccins oraux contre le choléra doivent être utilisées dans les situations d'urgence pour réduire la charge de morbidité.****

## Resumen

**Objetivo:**

Determinar si la vacuna oral preventiva contra el cólera reduce la gravedad de la enfermedad y la mortalidad en personas que desarrollan esta enfermedad durante un brote.

**Métodos:**

El estudio involucró un análisis retrospectivo de datos demográficos y clínicos de 41 instalaciones de tratamiento del cólera de Sudán del Sur en pacientes que desarrollaron la enfermedad entre el 23 de abril y el 20 de julio de 2014 durante un gran brote, pocos meses después de una campaña de vacunación oral preventiva. Los pacientes que desarrollaron deshidratación grave fueron diagnosticados con el cólera. Se comparó a los pacientes vacunados con los no vacunados y se utilizó un análisis de regresión logística multivariable para identificar los factores relacionados con el desarrollo de la enfermedad grave o la muerte.

**Resultados:**

En total, se trató a 4 115 pacientes con cólera en las 41 instalaciones: 1 946 (47,3%) sufrían de enfermedad grave y se produjeron 62 (1,5%) muertes. El análisis multivariable mostró que los pacientes que recibieron dos dosis de vacuna oral contra el cólera eran 4,5 veces menos propensos a desarrollar la enfermedad grave en comparación con los pacientes sin vacunar (coeficiente de posibilidades ajustado, CPa: 0,22; intervalo de confianza, IC, del 95%: 0,11–0,44). Asimismo, los pacientes con cólera grave tenían muchas más posibilidades de morir que los que no la padecían (CPa: 4,76; IC del 95%: 2,33–9,77).

**Conclusión:**

La vacunación preventiva con dos dosis de vacuna oral contra el cólera se relacionó con una importante reducción de las posibilidades de desarrollar la enfermedad grave del cólera durante un brote en Sudán del Sur. Asimismo, la enfermedad grave fue el mayor indicador de muerte. Deberían utilizarse dos dosis de vacuna oral contra el cólera en urgencias para reducir la carga de la enfermedad.

## ملخص

**الغرض:**

تحديد إذا ما كان التطعيم الفموي الوقائي ضد الكوليرا يحد من الآثار الوخيمة للمرض وحالات الوفاة الناتجة عنه لدى الأشخاص الذين يعانون من الإصابة بمرض الكوليرا، وذلك في فترة تفشي ذلك المرض.

**الطريقة:**

اشتملت الدراسة على تحليل بأثر رجعي للبيانات الديموغرافية والسريرية المستمدة من 41 منشأة لعلاج مرض الكوليرا في جنوب السودان فيما يتعلق بالمرضى الذين عانوا من الإصابة من مرض الكوليرا في الفترة بين 23 أبريل و20 يوليو 2014 في فترة الانتشار الواسع للمرض، الذي حدث بعد مرور أشهر معدودة على حملة التطعيم الفموي الوقائي. وتم اعتبار المرضى الذين عانوا من الإصابة بالجفاف الشديد ممن يعانون من الإصابة بعدوى شديدة لمرض الكوليرا. وجرت مقارنة المرضى الذين تلقوا التطعيم بالمرضى ممن لم يتلقوه، كما تم استخدام تحليل التحوف اللوجيستي متعدد المتغيرات لتحديد العوامل المرتبطة بالمعاناة من الآثار الوخيمة للمرض أو بحالات الوفاة الناتجة عنه.

**النتائج:**

إجماليًا، بلغ عدد المرضى المصابين بالكوليرا، ممن تلقوا العلاج في المنشآت المشار إليها والبالغ عددها 41 منشأة، 4115 مريضاً: 1946 (47.3%) ممن عانوا من آثار وخيمة للمرض و62 حالة وفاة (1.5%). وتبيّن من التحليل متعدد المتغيرات أن احتمال المعاناة من الآثار الوخيمة للمرض يقل بمعدل 4.5 مرات لدى المرضى الذين تلقوا جرعتين من التطعيم الفموي ضد الكوليرا مقارنةً بالمرضى الذين لم يتلقوا التطعيم (بنسبة احتمال معدّلة بلغت: 0.22؛ بنسبة أرجحية مقدارها ‏95‏%: 0.11–0.44). وعلاوةً على ذلك، زاد احتمال الوفاة إلى حد كبير لدى المرضى الذين عانوا من آثار وخيمة لمرض الكوليرا مقارنةً بهؤلاء المرضى الذين لم يعانوا من ذلك (بنسبة احتمال معدّلة بلغت: 4.76؛ ونسبة أرجحية مقدارها 95%: 2.33–9.77.).

**الاستنتاج:**

ثمة ارتباط بين تلقي جرعتين من التطعيم الوقائي للقاح الكوليرا الفموي والانخفاض الكبير في احتمالية المعاناة من الآثار الوخيمة لمرض الكوليرا في فترة تفشيه في جنوب السودان. وفضلًا عن ذلك، كانت الآثار الوخيمة للمرض أكثر العلامات إنذارًا بوقوع الوفاة. ويجب استخدام جرعتين من اللقاح الفموي ضد الكوليرا في الحالات العاجلة للتخفيف من عبء المرض. ****

## 摘要

**目的:**

旨在确定霍乱爆发时期，预防性口服霍乱疫苗能否降低霍乱患者的疾病严重性和死亡率。

**方法:**

本研究对南苏丹 41 家霍乱治疗机构中霍乱患者的人口学和临床数据进行了回顾性分析。这些霍乱患者在 2014 年 4 月 23 日至 7 月 20 日期间的霍乱大爆发中染病，疫情爆发前数月，该地曾开展过预防性口服霍乱疫苗活动。 表现出严重脱水症状的患者被视为严重霍乱感染。 我们对接受疫苗接种和未接受疫苗接种的患者进行了比较，并且使用多变量逻辑回归分析，以确定与感染严重霍乱或者死亡的相关因素。

**结果:**

这 41 家机构总共诊治了 4115 位霍乱患者： 1946 (47.3%) 例严重霍乱，62 (1.5%) 例死亡。 多变量分析显示接受两剂口服霍乱疫苗接种的患者比未接受疫苗接种的患者染上严重霍乱的可能性低 4.5 倍（调整比值比，aOR： 0.22；95% 置信区间，CI：0.11–0.44）。 此外，严重霍乱患者死亡的可能性远远高于非严重霍乱患者 (aOR: 4.76; 95% CI: 2.33–9.77)。

**结论:**

两剂预防性口服霍乱疫苗的接种与南苏丹霍乱爆发期间，感染严重霍乱的可能性显著降低有关。 此外，严重霍乱是死亡的最强预测指标。 应在紧急状态下使用两剂口服霍乱疫苗，以减轻疾病负担。

## Резюме

**Цель:**

Определить, способствует ли упреждающая пероральная вакцинация против холеры снижению тяжести заболевания и смертности среди людей, заболевших холерой во время вспышки.

**Методы:**

В ходе исследования был проведен ретроспективный анализ демографических и клинических данных из 41 центра лечения холеры в Южном Судане, относящихся к пациентам, заболевшим холерой в период между 23 апреля и 20 июля 2014 года во время масштабной вспышки спустя несколько месяцев после кампании предупреждающей пероральной вакцинации. Сильное обезвоживание у больных считалось признаком тяжелой формы холеры. Вакцинированных и невакцинированных пациентов сравнили, и с помощью множественной логистической регрессии были определены факторы, обуславливающие развитие тяжелой формы заболевания или смерть.

**Результаты:**

В общей сложности 4115 больных холерой проходили лечение в 41 центре: у 1946 (47,3%) наблюдалась тяжелая форма заболевания, в 62 (1,5%) случаях наступила смерть. Многомерный анализ показал, что вероятность развития тяжелой формы заболевания у пациентов, получивших две дозы пероральной противохолерной вакцины, была в 4,5 раза меньше, чем у невакцинированных пациентов (скорректированное отношение шансов, сОШ: 0,22; 95%-й доверительный интервал, ДИ: 0,11–0,44). Кроме того, у больных тяжелой формой холеры вероятность наступления смерти была гораздо выше, чем у пациентов с заболеванием меньшей степени тяжести (сОШ: 4,76; 95%-й ДИ: 2,33–9,77).

**Вывод:**

Упреждающая вакцинация двумя дозами пероральной противохолерной вакцины обусловила значительное уменьшение вероятности развития тяжелой формы холеры во время вспышки в Южном Судане. Кроме того, тяжелая форма заболевания являлась наиболее значимым фактором смерти. В чрезвычайных ситуациях для уменьшения бремени заболевания следует применять две дозы пероральной противохолерной вакцины.

## Introduction

Cholera is an extremely virulent diarrhoeal disease that affects both children and adults and can kill within hours if left untreated. Although the disease was largely eliminated from industrialized countries over a century ago by water and sewage treatment, it remains a major cause of morbidity and mortality in many areas of Africa and Asia. Every year, there are an estimated 3 to 5 million cases and 100 000 to 120 000 deaths due to cholera.^1^ Areas where minimum requirements for clean water and sanitation have not been met, such as peri-urban slums and camps for internally displaced people or refugees, are most at risk.^1^^,^^2^ Although the attack rate of cholera is high, fewer than 25% of those infected become ill.^1^ Among people who develop symptoms, 80% have mild or moderate disease, whereas around 20% develop acute watery diarrhoea with severe dehydration. As many as 80% can be treated successfully through prompt administration of oral rehydration salts and, with good or adequate fluid replacement (oral or intravenous), mortality is reduced to about 1%.^3^

The causative agent of cholera, *Vibrio cholerae*, is autochthonous to aquatic environments and cannot be eradicated. However, hydroclimatology-based prediction and prevention is an achievable goal. Access to clean water and adequate sanitation remain the mainstays of preventing both endemic cholera and cholera outbreaks, and health education can promote the adoption of appropriate hygiene practices.^4^ Cholera vaccination is increasingly being used as a safe and effective additional tool to supplement existing priority cholera control measures under the right conditions.^5^^,^^6^ In emergencies, vaccines provide immediate, short‐term protection while interventions to improve access to safe water and sanitation are put into place. Infectious disease can occur in previously vaccinated individuals: primary breakthrough infections are due to vaccine failure, whereas secondary breakthroughs are due to waning protective immunity. In such cases, the disease is usually milder than in the unvaccinated.^7^

Two oral cholera vaccines have been prequalified by the World Health Organization (WHO): Dukoral® and Shanchol™.^8^^,^^9^ Dukoral® provides 85–90% protection for 6 months in all age groups, whereas Shanchol™ ensures 65% protection for at least 5 years in individuals older than 1 year, both following two doses given at an appropriate interval. There is growing evidence that the vaccine also provides herd protection by interrupting disease transmission. A high level of immunization could, therefore, provide even greater protection to populations at risk.^10^ In total, more than 1.6 million doses of WHO prequalified oral cholera vaccine have been deployed in mass vaccination campaigns since 1997.^9^ In 2012, following the adoption of resolution WHA 64.15 by the 64th World Health Assembly in 2011,^8^ a WHO technical working group recommended that global cholera management should be boosted by creating a stockpile of 2 million doses of oral cholera vaccine for use in emergencies. This stockpile would help ensure that countries have rapid access to vaccine for cholera control.

The cholera vaccine stockpile was first used in 2014 in South Sudan, which peacefully seceded from Sudan in 2011 after 50 years of conflict. The country has a low level of physical, human and institutional development:^11^ in 2010, only 55% of the population had access to improved sources of drinking water and only 20% had access to a toilet facility.^12^ As a result, cholera is endemic. In December 2013, renewed fighting led to population displacement and dire overcrowding and inadequate sanitation for internally displaced people at the approach of the rainy season. This prompted the South Sudanese Ministry of Health, together with WHO and other partners, to implement a pre-emptive mass cholera vaccination campaign. When a cholera outbreak was declared in the country 5 months later, it was reported that displaced people living in makeshift camps at United Nations sites where the vaccination campaign took place were largely unaffected.^13^ However, it was not known whether the clinical characteristics or mortality of the disease was different in vaccinated people.

The aim of this study was, therefore, to investigate the effect of pre-emptive oral cholera vaccine on the severity of breakthrough disease and the case fatality rate by comparing vaccinated and unvaccinated patients who presented to health facilities in South Sudan. The setting was unusual because data were collected in the midst of an outbreak. In contrast, most previous studies were carried out in areas in which cholera was simply endemic.^5^^,^^10^^,^^14^^,^^15^

## Methods

The pre-emptive mass cholera vaccination campaign was implemented between February and April 2014 using Shanchol™. Vaccine coverage was between 60 and 85% across the country. Around 166 000 refugees and 40 000 of the host population, who were at a very high risk of morbidity and mortality, received the vaccine – at least 60 000 received two doses at an interval of 2 weeks or more.^13^^,^^16^^,^^17^ For this study, we retrospectively analysed clinical information from the 2014 South Sudan cholera data set on cholera patients seen at 41 cholera treatment facilities in nine affected counties in three states of the Equatoria Region between 23 April and 20 July 2014. We abstracted data on: (i) the patient’s sociodemographic characteristics, such as age, gender and place of residence; (ii) time of disease onset; (iii) time of arrival at the health facility; (iv) oral cholera vaccine status; (v) clinical signs and symptoms; (vi) laboratory test results; (vii) treatment; (viii) length of hospital stay; and (ix) disease outcome. In general, oral cholera vaccine status was determined from vaccination cards but, for patients who had misplaced their cards, status was self-reported and, if possible, verified by two family members.

The main outcome variables were: (i) death of a cholera case, which was categorized as a binary variable; and (ii) the severity of the cholera infection, which was determined from the depleted blood volume and expressed clinically as the degree of dehydration. Dehydration was assessed from blood pressure, the level of consciousness and skin turgor, among other criteria. Mild or moderate dehydration at entry to the health facility was considered to indicate simple cholera, whereas severe dehydration was classified as severe cholera or cholera gravis. The principle explanatory variable (i.e. the main exposure variable) was the number of oral cholera vaccine doses received. However, patients who received only one dose were not included in the analysis because of the small sample size. Other putative explanatory variables were sociodemographic characteristics, clinical assessment and treatment.

### Data analysis

The data set was checked for logical inconsistencies, invalid codes, omissions and improbable data by tabulating, summarizing, describing and plotting variables, depending on their nature. Missing observations were systematically excluded. In addition, cholera cases reported from the community were excluded because they constituted a small proportion of all cases and because exclusion made it easier to compare vaccinated and unvaccinated cases.

Summary statistics were presented as proportions for categorical variables, as means and standard deviations for normally distributed continuous variables and as medians and interquartile ranges (IQRs) for continuous variables with a skewed distribution. Associations between categorical variables were assessed using Pearson’s *χ*^2^ test or Fisher’s exact test for small samples, as appropriate. For continuous variables, mean differences between the two subgroups were assessed using Student’s *t* test. Associations between exposure variables and severe cholera and death were evaluated by a univariate logistic regression model; crude odd ratios, 95% confidence intervals (CIs) are reported. Subsequently, factors associated with severe cholera or death in the univariate analysis at a significance level below 5% were included in a multiple logistic regression model. Backward elimination based on a *P*-value lower than 0.05 was used to retain variables that were independently associated with severe cholera or death; the corresponding adjusted odds ratios (aORs) and 95% CIs for the final model are reported.^18^ Data analyses were performed using Stata version 13.1 (StataCorp. LP, College Station, United States of America) and Microsoft Excel 2013 (Microsoft Corporation, Redmond, USA) was used to plot the epidemic curve. Individual consent was not necessary because the study used existing clinical data and the identity of the patients was not disclosed. The study was approved by the Ministry of Health of the Republic of South Sudan.

## Results

In total, 4115 cholera patients were treated at the 41 cholera treatment facilities between 23 April and 20 July 2014. The results of cholera rapid diagnostic tests were available for 258 patients, of which 101 (39.1%) were positive. In addition, *V. cholerae* culture results were available for 31 patients, of which 22 (71.0%) were positive. However, only a few positive tests are necessary to confirm a cholera outbreak. Of the 4115 affected, 1907 (46.3%) were women and 900 (21.9%) were children younger than 5 years (Table 1). The median age of the patients was 20 years (IQR: 5–32). Eight patients (0.2%) had received one dose of oral cholera vaccine and 75 (1.8%) had received the recommended two doses.

**Table 1:** Baseline characteristics, cholera cases at 41 treatment facilities in three states, South Sudan, 2014

Baseline characteristic	No. of cholera cases^a^ (%) (*n* = 4115)
**Sex**	
Female	1907 (46.3)
Male	2207 (53.6)
**Age, years**	
< 5	900 (21.9)
5–49	2879 (70.0)
≥ 50	312 (7.6)
**Oral cholera vaccine doses, no.**	
0	4019 (97.7)
1	8 (0.2)
2	75 (1.8)
**Phase of cholera epidemic**	
First wave (weeks 17–24 of 2014)	1735 (42.2)
Second wave (weeks 25–30 of 2014)	2380 (57.8)
**Reporting health facility**	
Local health facility	3142 (76.4)
MSF health facility	972 (23.6)
**County**	
Ikotos	3 (0.1)
Juba	2077 (50.5)
Kajo Keji	60 (1.5)
Kopoeta North	51 (1.2)
Lopa-Lafon	94 (2.3)
Magwi	164 (4.0)
Mundri East	3 (0.1)
Torit	1614 (39.2)
Yei	48 (1.2)
**Delay before presentation, days**	
≤ 1	3889 (94.5)
> 1	206 (5.0)
**Nature of diarrhoea**	
Clear-water stools	1970 (47.9)
Rice-water stools	2037 (49.5)
**Vomiting**	
No	341 (8.3)
Yes	3625 (88.1)
**Degree of dehydration**	
Mild	1017 (24.7)
Moderate	1067 (25.9)
Severe	1946 (47.3)
**Hospitalization**	
No	135 (3.3)
Yes	3978 (96.7)
**Outcome**	
Discharged alive	2931 (71.2)
Died	62 (1.5)
Still hospitalized	297 (7.2)
Self-discharged	30 (0.7)
Readmitted	15 (0.4)

MSF: Médecins Sans Frontières.

^a^ As the information on some variables was missing for some patients, the figures do not always add up to 100%.

**Fig. 1:**
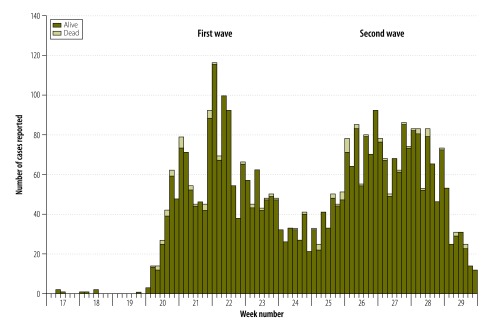
Cholera cases reported at 41 treatment facilities in three states, South Sudan outbreak, 2014

The first case was a 28-year-old man who had received a single dose of oral cholera vaccine and who attended a Médecins Sans Frontières clinic at a camp for internally displaced people in Juba County. Almost 90% of the 4115 patients were observed in two of the nine study counties: 2077 patients (50.5%) in Juba County and 1614 (39.2%) in Torit County (Table 1). The attack rate could not be determined because not all cases were identified. Local health facilities treated 3142 patients (76.4%) – the others were treated in facilities run by Médecins Sans Frontières. The epidemic curve (Fig. 1) depicts the number of cases reported daily between the start of the outbreak in the 17th week of 2014 and the 29th week. The epidemic propagated in two distinct waves: the first wave occurred before the 25th week and the second, after the 25th week. Most cases, namely 2380 (57.8%), occurred in the second wave. The mean time from disease onset to presentation at a health facility was 0.4 days, although 206 patients (5.0%) sought care more than 1 day after onset.

Of the 4115 cholera patients, 1946 (47.3%) were severe (Table 1). Three of the eight patients (37.5%) who had received one vaccine dose were severe, as were 12 of 74 (16.2%) who had received two doses. After controlling for confounding, multivariate analysis showed that the odds of developing severe disease was around 4.5-fold lower in patients who had received two vaccine doses than in unvaccinated patients (aOR: 0.22; 95% CI: 0.11–0.44; Table 2). Other factors independently linked to lower odds of severe disease included: (i) presentation in the second wave of the epidemic (aOR: 0.43; 95% CI: 0.36–0.53); and (ii) the absence of multiple symptoms (aOR: 0.13; 95% CI: 0.10–0.15). Factors independently associated with higher odds of severe cholera were: (i) presentation more than 1 day after disease onset (aOR: 2.33; 95% CI: 1.53–3.55); (ii) rice-water stools (aOR: 3.25; 95% CI: 2.66–3.96); and (iii) vomiting (aOR: 6.38; 95% CI: 4.28–9.52).

**Table 2:** Factors associated with severe cholera, South Sudan outbreak, 2014

Risk factor	No. of all cholera cases^a^	No. of severe cholera cases (%)^b^	Risk of severe cholera	
			Univariate analysis	Multivariate analysis
			OR (95% CI)	aOR (95% CI)
**Oral cholera vaccine doses, no.**				
0	3939	1926 (48.9)	Reference	Reference
2	74	12 (16.2)	0.20 (0.11–0.38)	0.22 (0.11–0.44)
**Phase of cholera epidemic**				
First wave (weeks 17–24 of 2014)	1733	1239 (71.5)	Reference	Reference
Second wave (weeks 25–30 of 2014)	2295	707 (30.8)	0.18 (0.15–0.20)	0.43 (0.36–0.53)
**Delay before presentation, days**				
≤ 1	3808	1801 (47.3)	Reference	Reference
> 1	203	133 (65.5)	2.12 (1.57–2.85)	2.33 (1.53–3.55)
**Nature of diarrhoea**				
Clear-water stools	1900	418 (22.0)	Reference	Reference
Rice-water stools	2030	1508 (74.3)	10.24 (8.84–11.87)	3.25 (2.66–3.96)
**Vomiting**				
No	340	34 (10.0)	Reference	Reference
Yes	3588	1898 (52.9)	10.12 (7.06–14.51)	6.38 (4.28–9.52)
**Multiple symptoms^c^**				
Yes	2328	1697 (72.9)	Reference	Reference
No	1442	186 (12.9)	0.06 (0.05–0.07)	0.13 (0.10–0.15)

aOR: adjusted odds ratio; CI: confidence interval; OR: odds ratio.

^a^ All cholera cases reported at 41 treatment facilities in three states for whom relevant data were available.

^b^ Percentage of all cholera cases.

^c^ Multiple symptoms included abdominal, leg or arm cramps, body weakness and headache.

Of the 4115 patients with cholera disease, 62 (1.5%) died (Table 1). None of the eight patients who received one vaccine dose died. The main risk factor for death was severe disease, which was associated with nearly a fivefold increase in odds compared with mild or moderate disease (aOR: 4.76; 95% CI: 2.33–9.77; Table 3). The odds of death was also raised in patients aged 50 years or more compared with younger adults (aOR: 3.42; 95% CI: 1.65–7.08). In contrast, hospitalization was associated with a significantly lower odds of death (aOR: 0.08; 95% CI: 0.03–0.19).

**Table 3:** Factors associated with death due to cholera, South Sudan outbreak, 2014

Risk factor	No. of all cholera cases^a^	No. of deaths (%)^b^	Risk of death	
			Univariate analysis	Multivariate analysis
			OR (95% CI)	aOR (95% CI)
**Age, years**				
< 5	700	14 (2.0)	1.44 (0.77–2.71)	1.17 (0.85–3.40)
5–49	2428	34 (1.4)	Reference	Reference
≥ 50	245	12 (4.9)	3.55 (1.81–6.95)	3.42 (1.65–7.08)
**Cholera severity**				
Mild or moderate	1571	11 (0.7)	Reference	Reference
Severe	1720	43 (2.5)	3.62 (1.86–7.04)	4.76 (2.33–9.77)
**Hospitalization**				
No	87	11 (12.6)	Reference	Reference
Yes	3188	51 (1.6)	0.11 (0.06–0.22)	0.08 (0.03–0.19)

aOR: adjusted odds ratio; CI: confidence interval; OR: odds ratio.

^a^ All cholera cases reported at 41 treatment facilities in three states for whom relevant data were available.

^b^ Percentage of all cholera cases.

## Discussion

We found that pre-emptive vaccination with two doses of oral cholera vaccine was associated with a 4.5-fold reduction in the likelihood of developing severe cholera during an outbreak in South Sudan compared with no vaccination. We were unable to determine whether a single dose was protective against severe disease because few vaccinated cholera patients had received only one dose. There is thus still a need to investigate whether a single dose may offer cost-effective protection. As the current recommendation for effective disease prevention is two doses of oral cholera vaccine,^19^ it is likely that two doses are required to reduce disease severity in vaccinees who develop clinical disease. Consequently, vulnerable communities should be persuaded to accept and adhere to multiple vaccination campaigns. Our study findings suggest that mass cholera vaccination could be useful in emergency settings or with displaced populations where cholera outbreaks are a likely occurrence. More studies to understand the cost–effectiveness of these interventions are needed.^15^

Our findings contrast with those of an Indian study^14^ which failed to show that oral cholera vaccine mitigated against severe disease though there was some evidence of protection against clinical disease. Although the results from this and our study – whether oral cholera vaccine can reduce disease severity – are inconsistent, the beneficial effect of vaccines on the severity of other infectious diseases has been extensively described.^7^ Milder disease in vaccinees has been reported with the rotavirus vaccine against rotavirus diarrhoeal disease,^20^ the pertussis vaccine,^21^ the varicella vaccine^22^ and the bacille Calmette–Guérin vaccine against tuberculosis.^23^

We found that the burden of severe disease was greatest in the first half of the outbreak. Consequently, control measures must be put in place as soon as possible during an outbreak; the prepositioning of vaccine stocks in areas prone to epidemics would help. In our study, patients who attended treatment centres more than 24 hours after disease onset were at a greater risk of severe cholera. Raising awareness, case-finding, tracing contacts and setting up treatment facilities close to affected populations would reduce the delay in seeking care. Patients who presented with vomiting or rice-water stools were more likely to suffer severe illness. The presence of rice-water stools may suggest a high bacterial load and should be monitored closely. Patients with vomiting should be admitted for rehydration with intravenous fluids since oral treatment may be ineffective. Almost half the patients in our study presented with severe illness, perhaps because milder cases were less likely to attend the health facilities from which we obtained our data.

The case fatality rate in our study was 1.5%, which is high given that the benchmark for optimal care is below 1%. However, subsequent reports from South Sudan gave a rate of 2.26% in October 2014.^24^ In recent years, the highest case fatality rate in the country was 2.9% in 2006. Between 2007 and 2013, the case fatality rate ranged from 0.07% to 1.8%.^25^ More recently, the 2015 outbreak had a case fatality rate of 2.6%.^26^ Overall, rates in South Sudan are similar to those reported in Africa as a whole, which average 2%,^27^ and to those observed during earlier conflicts in the same region.^28^^–^^30^ We were unable to analyse the direct effect of the oral cholera vaccine on death due to cholera because there was only one death among vaccinees who developed the disease. However, since death in cholera results from severe volume depletion and since, in our study, severe dehydration (i.e. severe disease) was the greatest risk factor for death, it is reasonable to conclude that the oral cholera vaccine reduced the number of deaths not only by preventing clinical disease but also by protecting the vaccinated against severe disease.

Cholera is distinctive among diarrhoeal diseases in that mortality is high among patients of all ages in the absence of treatment, though a case fatality rate under 1% can be achieved even in makeshift treatment centres.^31^^–^^33^ We found that patients aged 50 years and more are particularly vulnerable in a humanitarian crisis, as are children younger than 5 years and pregnant women. Today, as a consequence of the Sphere Project, which advocates minimum standards for a humanitarian response,^2^ humanitarian relief has improved and outcomes after cholera outbreaks are no longer so far below optimal. Any delay in providing adequate rehydration therapy can result in rapid dehydration, even in patients initially classified as having mild disease. In our study, 11 patients with mild or moderate cholera died. Our data identified hospitalization as strongly associated with an increased chance of survival. Although not all cholera patients must be hospitalized, hospitalization may be an option for all patients in a conflict setting where movement is restricted by insecurity, as occurred in South Sudan. 

Our study had several limitations. The overall picture of the cholera outbreak was incomplete because our data did not cover cases in the community, the outcomes of cases still being treated in facilities during data collection or cases that occurred after data collection. Moreover, missing observations may have been subject to recall bias and there was no comparison group. As a result, the case fatality rate may have been underestimated. Neither the attack rate nor the risk of acquiring cholera could be calculated because an adequate denominator was lacking. Moreover, the study lacked the power to determine whether the oral cholera vaccine had an effect on mortality. The observational design meant that it was possible to demonstrate only an association between vaccination and cholera severity and not a causal relationship. However, the strength of the association, the temporal sequence between vaccination and the cholera outbreak, the biological plausibility of the action of the vaccine and the consistency of our findings with current knowledge all suggest that the oral cholera vaccine reduced the burden of cholera during the outbreak. Our assessment of the degree of dehydration was based on indicators that may have been open to subjective interpretation. Nevertheless, patients were diagnosed by trained medical personnel in clinical facilities. In the absence of vaccination cards, information about vaccination status was obtained from the patients themselves, which increased the risk of reporting bias. However, efforts were made to verify vaccination status with family members.

In conclusion, this study indicates that two pre-emptive doses of oral cholera vaccine can reduce the severity of cholera disease among vaccinees during an outbreak and thereby reduce the disease burden. Importantly, our data were collected during an outbreak and not simply in an endemic setting. It should be recognized that two doses of oral cholera vaccine are probably necessary and that oral cholera vaccine campaigns should include two rounds. Moreover, oral cholera vaccine should be used in emergencies to reduce the disease burden.
